# Mechanisms of inflammatory cell activation in periodontitis-induced bone destruction and advances in targeted interventions

**DOI:** 10.3389/fimmu.2026.1891787

**Published:** 2026-09-29

**Authors:** Ying Zhuang, Yingzi Zhang, Weihai Xu

**Affiliations:** 1 Department of Stomatology, Binhai County People’s Hospital, Yancheng, Jiangsu, China; 2 Department of Ophthalmology, Binhai County People’s Hospital, Yancheng, Jiangsu, China

**Keywords:** bone destruction, inflammatory cell activation, macrophage polarization, osteoclast, periodontitis, targeted therapy

## Abstract

Periodontitis is a leading cause of tooth loss in adults, characterized primarily by alveolar bone destruction driven by dysregulated host immune-inflammatory responses. Central to this pathological process is the aberrant activation of inflammatory cells within the periodontal microenvironment. This review comprehensively summarizes the activation phenotypes, functions, and interaction networks of key inflammatory cells including macrophages, neutrophils, T cells, and B cells in periodontitis. It elucidates the molecular and cellular mechanisms by which these activated cells contribute to bone destruction through the release of inflammatory mediators and regulation of osteoclastogenesis and osteoclast function. Furthermore, the review highlights recent advances in targeted therapeutic strategies aimed at modulating inflammatory cell activation, encompassing small molecule inhibitors, biologics, and cell-based therapies. By integrating current knowledge on inflammatory cell dynamics and targeted interventions. This review aims to identify key therapeutic targets within inflammatory cell networks to guide the development of precision therapies for periodontitis.

## 1 Introduction

Periodontitis is a chronic inflammatory disease initiated by the accumulation of bacterial plaque biofilms on the tooth surface, leading to progressive destruction of the periodontal supporting tissues, including the alveolar bone. Traditionally, the pathogenesis of periodontitis was explained by the non-specific plaque hypothesis, which emphasized the direct pathogenic effects of microbial biofilms. However, this concept has evolved substantially with the recognition that the host immune response plays a pivotal role in disease progression. The current understanding highlights that dysregulated host immune and inflammatory responses to periodontal pathogens are central to the tissue destruction observed in periodontitis (1). This shift from a purely microbial-centric view to a host-microbe interaction model underscores the complexity of the periodontal microenvironment, where innate and adaptive immune cells are persistently activated, orchestrating a network of cytokines, chemokines, and enzymes that collectively drive inflammation and bone resorption.

The alveolar bone is a highly dynamic tissue characterized by rapid remodeling, which is tightly regulated by the balance between osteoclast-mediated bone resorption and osteoblast-mediated bone formation. In periodontitis, this balance is disrupted, favoring osteoclastic activity and resulting in pathological bone loss (2). The alveolar bone’s unique anatomical and functional features, including its close association with teeth and exposure to oral pathogens and mechanical stress, contribute to its susceptibility to inflammatory insults. Recent studies have elucidated the role of inflammasomes, particularly the Nod-like receptor family pyrin domain containing 3(NLRP3) inflammasome, in mediating inflammatory responses that exacerbate alveolar bone destruction. Inflammasome activation leads to the maturation and secretion of pro-inflammatory cytokines such as Interleukin-1 beta (IL-1β) and Interleukin-18(IL-18), and induces pyroptosis, a form of inflammatory cell death, thereby amplifying local inflammation and osteoclastogenesis. Dysregulated inflammasome activity has been implicated not only in periodontitis but also in other alveolar bone diseases, highlighting its central role in osteoimmunology.

In the context of periodontitis, various immune cells including macrophages, neutrophils, T helper 17 (Th17) cells, and osteocytes contribute to the inflammatory milieu and bone resorption. For instance, osteocytes, traditionally viewed as mechanosensors, have been shown to directly regulate osteolysis through Myeloid Differentiation Primary Response Gene 88(MYD88)-dependent signaling pathways in response to bacterial pathogen-associated molecular patterns (PAMPs), promoting Receptor Activator of Nuclear Factor-κB Ligand (RANKL) expression and osteoclast activation (3). Similarly, Th17-derived cytokine IL-17A plays a dual role by defending against pathogens but also by exacerbating inflammation and bone loss when hyperactivated. Targeting IL-17A with sustained local delivery of neutralizing antibodies has demonstrated efficacy in limiting inflammatory bone loss in experimental periodontitis models (1). These findings illustrate the multifaceted roles of immune cells and their mediators in periodontal bone destruction.

Oxidative stress and mitochondrial dysfunction have emerged as critical factors in the regulation of osteoclastogenesis and alveolar bone resorption. The mitochondrial kinase Phosphatase and Tensin Homolog (PTEN) induced putative kinase 1(PINK1), essential for mitophagy and mitochondrial quality control, restrains excessive osteoclast differentiation and bone loss in periodontitis by mitigating mitochondrial reactive oxygen species (ROS) accumulation (4). Conversely, elevated ROS levels promote osteoclastogenesis and inflammatory osteolysis, and antioxidant strategies such as ROS-responsive delivery of resveratrol have shown promise in reducing bone destruction (5). These insights highlight the interplay between cellular metabolism, oxidative stress, and immune activation in periodontal bone pathology.

The aging process further complicates the immune and bone remodeling dynamics in periodontitis. Aging-associated immunosenescence and chronic low-grade inflammation, termed inflammaging, skew the immune environment towards a pro-inflammatory state, exacerbating alveolar bone loss and impairing regeneration (6). This underscores the need for tailored therapeutic approaches that consider the altered immune landscape in elderly patients.

Given the central role of inflammation and immune cell activation in alveolar bone destruction, targeted interventions aimed at modulating specific inflammatory pathways and cellular players have gained attention. Therapeutic strategies include inhibition of key signaling molecules such as Nuclear Factor Kappa-B (NF-κB), Mitogen-activated protein kinase (MAPK), and inflammasome components, as well as modulation of osteoclast differentiation and function through agents like protocatechualdehyde, kurarinone, and parthenolide. Moreover, specialized pro-resolving lipid mediators (SPMs) such as resolvins and maresins have demonstrated efficacy in promoting resolution of inflammation and enhancing bone regeneration in preclinical models (7). The development of novel drug delivery systems, including local sustained release formulations, further enhances the therapeutic potential of these agents by ensuring targeted and controlled modulation of the periodontal microenvironment (1).

In addition to pharmacological interventions, lifestyle factors and systemic conditions influence periodontal inflammation and bone loss. For example, metabolic syndrome and obesity exacerbate periodontal disease severity through systemic inflammation and altered immune responses. Conversely, voluntary physical activity has been shown to mitigate alveolar bone loss by modulating inflammatory cytokine expression (8). The bidirectional relationship between periodontitis and systemic diseases such as osteoporosis and rheumatoid arthritis further emphasizes the importance of integrated management strategies.

Advances in high-throughput technologies, including single-cell sequencing and lineage tracing, have provided deeper insights into the heterogeneity and dynamic activation states of inflammatory cells in periodontitis. These approaches facilitate the identification of specific cell subsets and molecular pathways that drive bone destruction, enabling the development of precision medicine approaches (2). Furthermore, the establishment of comprehensive multimodal datasets integrating clinical, radiographic, and molecular data supports the advancement of artificial intelligence-driven diagnostic and prognostic tools for periodontal disease (9).

Despite these advances, the dynamic interplay and temporal activation of macrophages, neutrophils, T cells, and B cells in driving osteoclastogenesis remain insufficiently integrated. In addition, few reviews have directly connected inflammatory cell phenotypes with emerging therapeutic targets. This review therefore aims to systematically synthesize recent findings on the activation networks of key inflammatory cells. In summary, periodontitis is a complex inflammatory disease characterized by dysregulated activation of innate and adaptive immune cells, leading to a pro-inflammatory and pro-osteoclastic microenvironment that drives alveolar bone destruction. Understanding the molecular and cellular mechanisms underlying inflammatory cell activation and bone remodeling dysregulation has paved the way for novel targeted therapeutic interventions. Continued research integrating immunology, bone biology, and advanced technologies holds promise for improving the prevention and treatment of periodontitis and its associated bone loss.

Periodontal dysbiosis is characterized by a microbial community shift in subgingival biofilms, favoring Gram-negative anaerobic pathobionts such as Porphyromonas gingivalis, Tannerella forsythia, and Treponema denticola. These keystone pathogens display pathogen-associated molecular patterns (PAMPs)—including lipopolysaccharide (LPS), fimbriae, and bacterial DNA—which are recognized by pattern recognition receptors (PRRs) such as Toll-like receptors (TLRs) on gingival epithelial cells, fibroblasts, and innate immune cells. This PAMP-PRR engagement triggers intracellular signaling cascades (e.g., NF-κB, MAPK) that drive the production of pro-inflammatory cytokines (IL-1β, Interleukin-6(IL-6), TNF-α) and the recruitment of neutrophils, macrophages, and dendritic cells. Although the retrieved literature is based on infected cutaneous wound models, it supports these conserved innate immune mechanisms: LPS-stimulated macrophages produce inflammatory mediators, and this response can be modulated by biomaterials that promote M2-like reparative polarization and reduce reactive oxygen species (ROS) and nitric oxide (10). Another hydrogel system combining nitric oxide release and antibiotic delivery reduced IL-1β and increased IL-10 while scavenging ROS in infected wounds, thereby dampening the inflammatory cascade (11) Similarly, a polymerizable ionic liquid-based hydrogel drove macrophage differentiation toward an M2 (reparative) phenotype and suppressed TNF-α and IL-6 while maintaining antibacterial activity (12). These approaches exemplify the “biological” antibacterial strategy, in which hydrogels modulate the host microenvironment and immune response rather than directly killing bacteria (13). In a diabetic wound model, a pH-responsive multifunctional hydrogel further accelerated healing by shifting macrophages from the pro-inflammatory M1 phenotype to the M2 phenotype, inhibiting inflammation, stimulating angiogenesis, and enhancing collagen deposition (14). While these studies confirm general cytokine networks (IL-1β, TNF-α, IL-6, Interleukin-10(IL-10)) and macrophage polarization pathways shared with periodontal pathology, they do not directly address the periodontal-specific RANKL/RANK/OPG (osteoprotegerin) axis or osteoclast-mediated alveolar bone resorption. Nevertheless, the upstream immune activation and inflammatory milieu described in these reports are integral components of the host response that, in periodontitis, ultimately drives alveolar bone destruction. Here, we provide a diagram (**Figure 1**) as an overview of the Immune mechanisms related to bone destruction.

**Figure 1:**
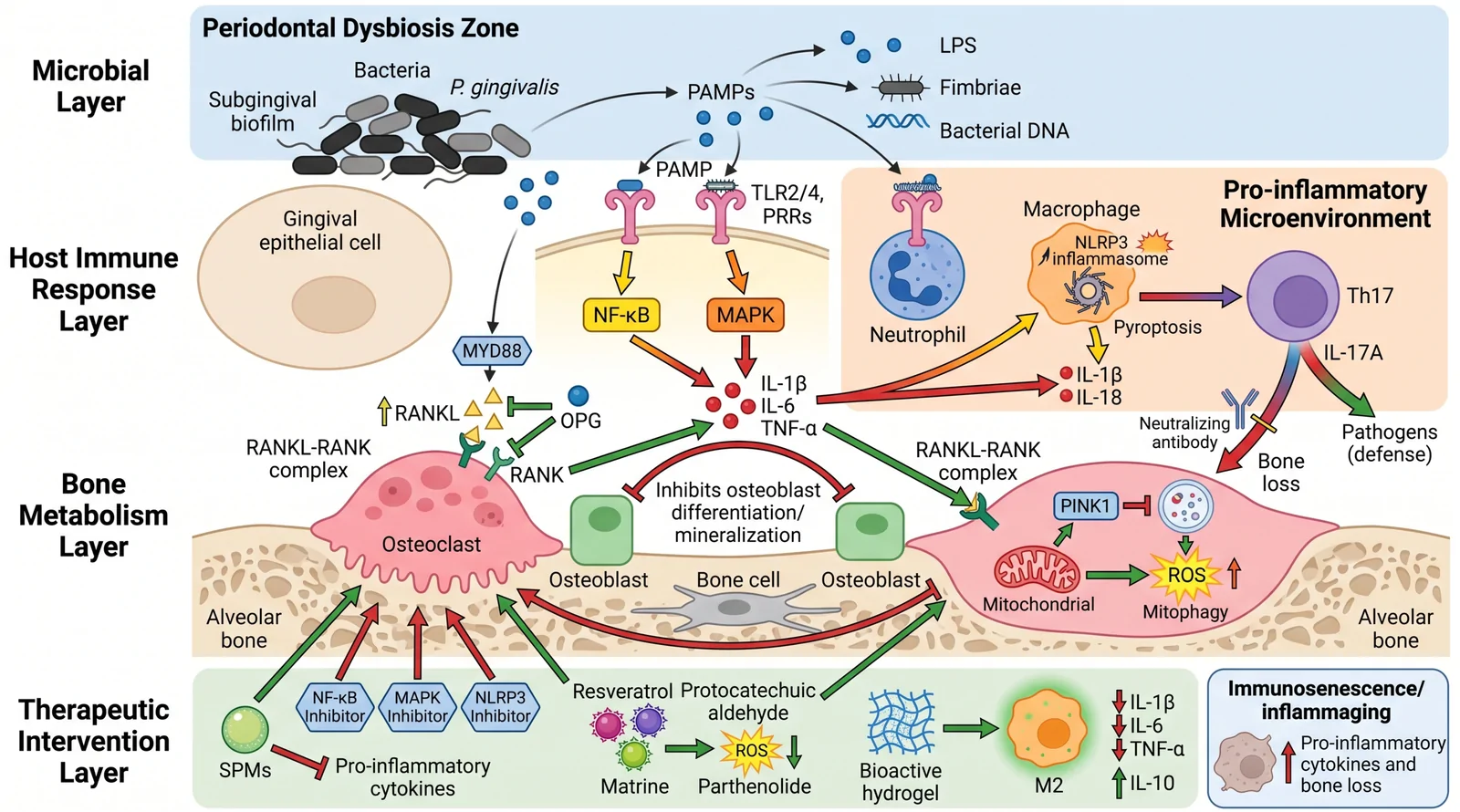
Immune mechanisms related to bone destruction.

## 2 Key inflammatory cell activation phenotypes and functions in the periodontitis microenvironment

### 2.1 Macrophage polarization and functional conversion

In the complex microenvironment of periodontitis, macrophages exhibit remarkable plasticity, undergoing polarization into distinct functional phenotypes that critically influence disease progression and tissue remodeling. Macrophages exhibit remarkable plasticity and can adopt diverse activation states along a functional spectrum. Although they are often described as pro-inflammatory ‘M1-like’ or anti-inflammatory, tissue-repairing ‘M2-like’ phenotypes, single-cell analyses have revealed substantially greater heterogeneity in periodontitis lesions. In periodontitis, stimuli such as bacterial lipopolysaccharide (LPS) and inflammatory cytokines drive macrophages from a resting M0 state toward M1 or M2 polarization. M1 macrophages are characterized by high expression of inducible nitric oxide synthase (iNOS), tumor necrosis factor-alpha (TNF-α), interleukin (IL)-1β, and IL-6, which collectively orchestrate robust inflammatory responses and promote alveolar bone resorption. M1 macrophages promote osteoclast formation and bone resorption function directly or indirectly by secreting mediators such as TNF-α, IL-1β, IL-6, and Nitric Oxide (NO), and relying on key signaling pathways such as NF-κB and Signal Transducers and Activators of Transcription 1(STAT1).Clinical and experimental studies have demonstrated an increased M1/M2 ratio in severe chronic periodontitis, indicating a skewing toward the pro-inflammatory phenotype that exacerbates periodontal tissue destruction (15). Recent advances using single-cell RNA sequencing have revealed a more nuanced heterogeneity within macrophage populations in periodontitis, including identification of osteoclastogenic subpopulations expressing high levels of Spp1 (osteopontin) and matrix metalloproteinase 9 (MMP9), which are closely associated with bone resorption sites, highlighting their direct contribution to alveolar bone loss (16). Conversely, M2 macrophages, which secrete anti-inflammatory cytokines such as IL-10 and transforming growth factor-beta (TGF-β), increase during disease remission or following treatment, facilitating resolution of inflammation and tissue repair. However, in chronic inflammatory settings, M2 macrophage function may become dysregulated, manifesting as inadequate inflammation clearance and impaired bone repair due to deficiency, as well as immune suppression and osteoclast activation caused by excessive M2 polarization, thereby promoting bone destruction (17). Innovative therapeutic strategies targeting macrophage polarization have emerged, exemplified by novel nanodrugs designed to selectively inhibit M1 macrophage inflammatory pathways, such as the Glucose-Polyethylene Glycol-Poly(lactic-co-glycolic acid) loaded with mCC950 (glucose-PEG-PLGA@MCC950) nanodrug that targets Glucose Transporter 1 (GLUT1)-expressing M1 macrophages to suppress NLRP3 inflammasome activation, thereby reducing periodontal tissue damage and promoting M2-associated anti-inflammatory responses (18). Moreover, interferon-beta (IFNβ) has been implicated in modulating macrophage phenotypes in periodontitis, promoting M1-to-M2 transition and enhancing IL-10 production via Interferon-stimulated gene 15(ISG15) induction, which contributes to immunosuppression and tissue homeostasis restoration (19). Collectively, these findings underscore the pivotal role of macrophage polarization dynamics in the pathogenesis and resolution of periodontitis, suggesting that therapeutic modulation of macrophage phenotypes represents a promising avenue for controlling periodontal inflammation and preventing bone destruction. And the **Figure 2** shows the macrophage polarization and functional conversion.

**Figure 2:**
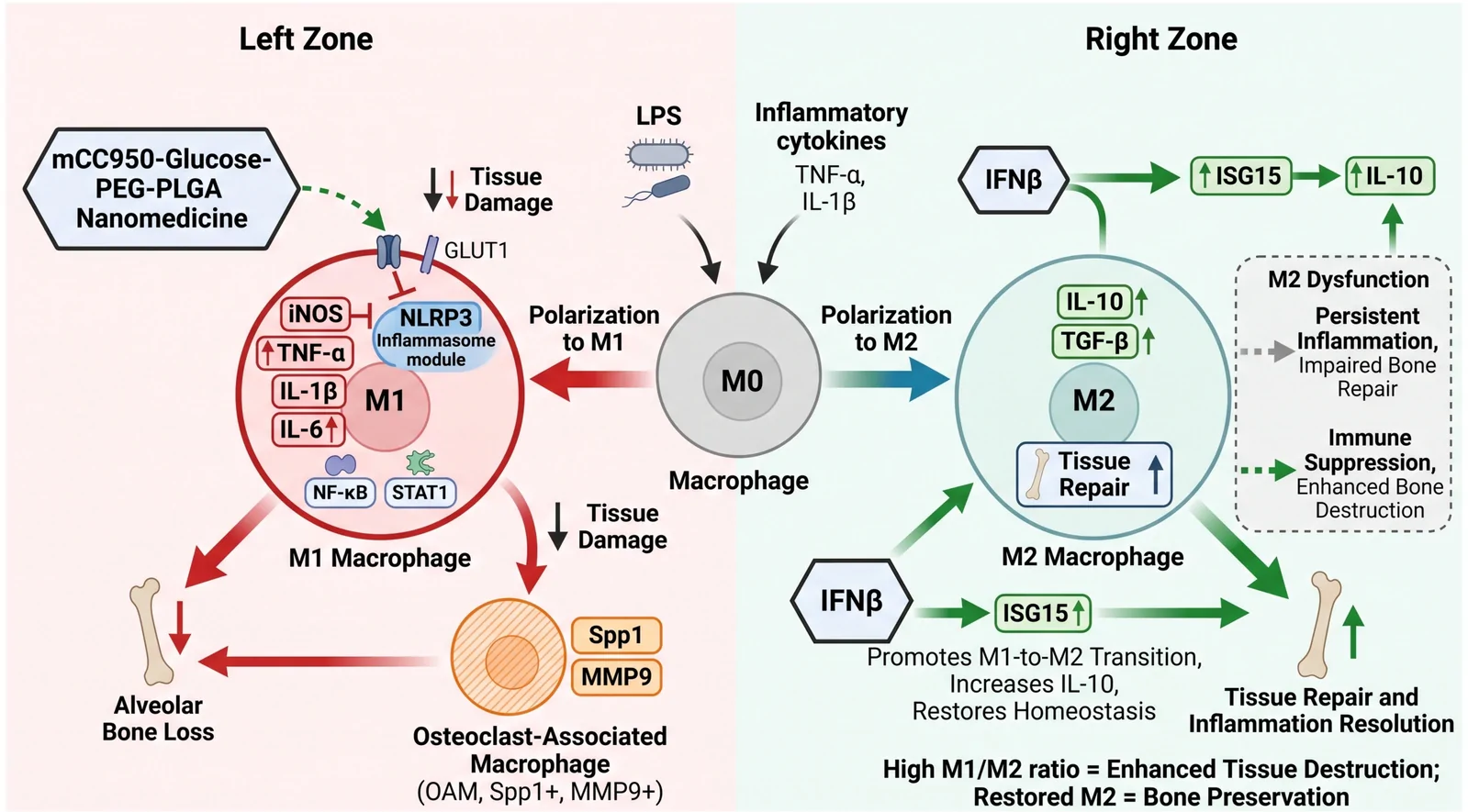
Macrophage polarization and functional conversion.

### 2.2 Neutrophil overactivation and NETosis

Neutrophils serve as the frontline defenders against periodontal pathogens, rapidly migrating to sites of infection to execute antimicrobial functions. However, in periodontitis, excessive neutrophil activation contributes to collateral host tissue damage, a phenomenon often referred to as “bystander injury.” Reactive oxygen species (ROS) has a bidirectional effect on neutrophil function: moderate levels of ROS are necessary for phagocytosis and bacterial killing (1), but excessive ROS release may damage adjacent tissues. Therefore, the initiation process is crucial in regulating the intensity of ROS production, enabling neutrophils to mount a more robust respiratory burst in response to subsequent stimuli, thereby balancing host defense and inflammatory injury (20). A hallmark of neutrophil activation in periodontitis is the formation of neutrophil extracellular traps (NETs), web-like structures composed of decondensed chromatin decorated with histones and granular proteins. While NETs effectively trap and kill bacteria, their components, particularly histones and proteases, possess potent cytotoxicity toward periodontal ligament cells and osteoblasts, directly impairing tissue integrity and promoting osteoclastogenesis (21). Recent investigations have revealed that neutrophils in periodontitis patients exhibit “hyperresponsive state” or “primed” state” characterized by enhanced antimicrobial and pro-inflammatory responses upon repeated pathogen exposure, which may underlie the persistence of chronic inflammation (22). Quantitative analyses demonstrate elevated neutrophil counts and increased chemotactic activity in periodontitis compared to healthy controls, correlating positively with clinical parameters such as probing depth and bleeding indices (23). Moreover, salivary neutrophil levels decrease significantly following periodontal therapy, indicating their potential utility as non-invasive biomarkers for monitoring disease activity and treatment efficacy (24). The dualistic role of neutrophils as both protectors and perpetrators in periodontal disease highlights the necessity for therapeutic approaches that modulate neutrophil activation and NETosis to mitigate tissue damage while preserving antimicrobial defense. Here, we proposed a working model (**Figure 3**) in which we could understand the Neutrophil Overactivation and NETosis.

**Figure 3:**
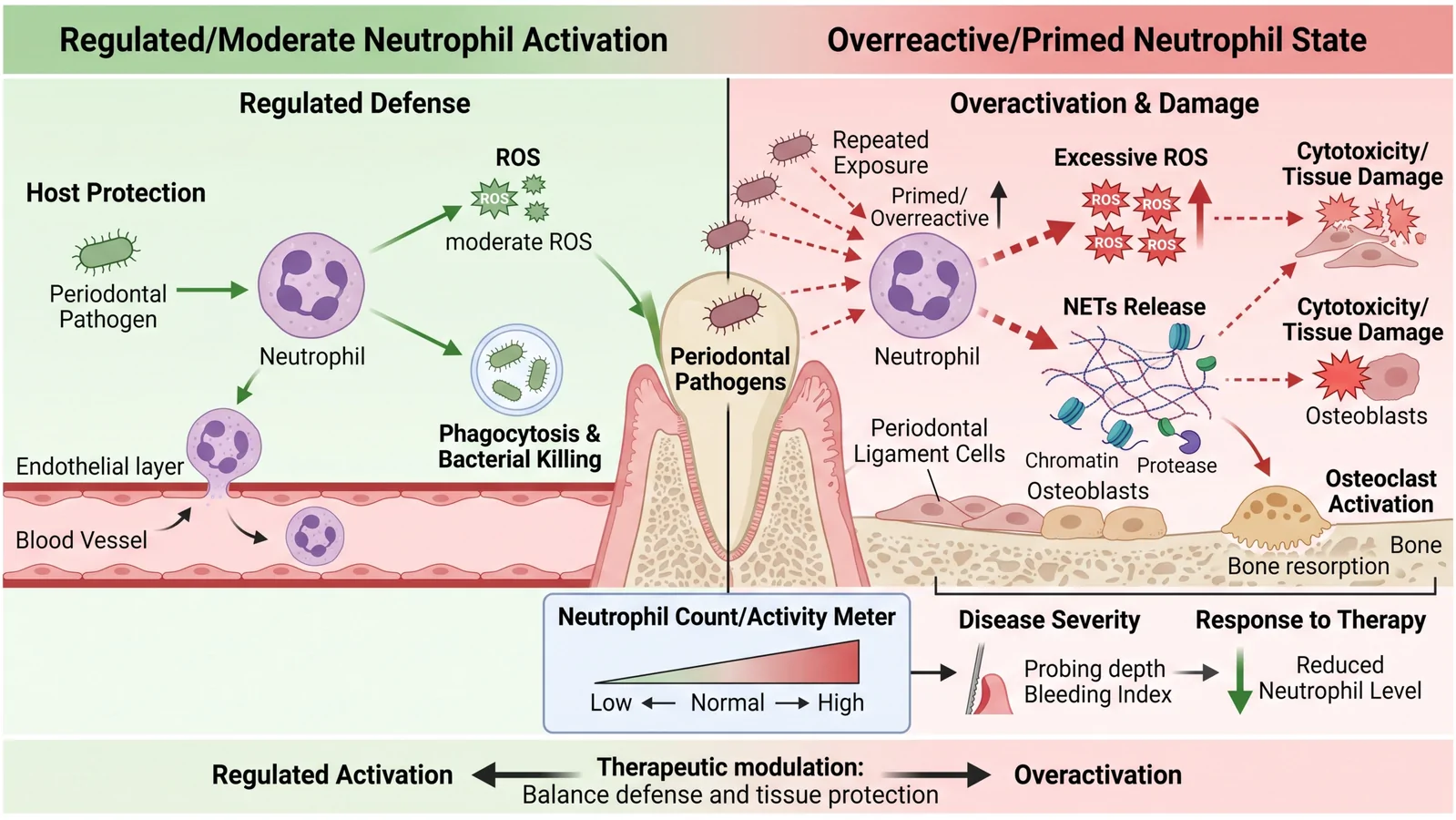
Neutrophil overactivation and NETosis.

### 2.3 Activation and regulation of adaptive immune cells (T cells and B cells)

Adaptive immunity, mediated by T and B lymphocytes, plays a critical role in orchestrating the immune response in periodontitis, influencing both inflammation and bone remodeling. Among T cell subsets, Th17 cells are key drivers of alveolar bone destruction, with Th1 cells also contributing to the inflammatory milieu. Th17 cells secrete IL-17A and IL-17F, potent pro-inflammatory cytokines that recruit and activate neutrophils and stimulate fibroblasts and osteoblasts to express receptor activator of nuclear factor kappa-β ligand (RANKL), thereby amplifying osteoclastogenesis and bone resorption (25). Elevated Th17 cell frequencies and increased IL-17 levels have been documented in both chronic and aggressive periodontitis, correlating with disease severity (26). Conversely, regulatory T cells (Tregs) maintain immune tolerance and limit bone loss by suppressing excessive inflammation. However, in active periodontitis, Treg numbers and function are often impaired, leading to an imbalance favoring Th17 responses and unchecked inflammation (27). Intriguingly, Tregs in periodontitis may exhibit plasticity, acquiring Th17-like characteristics, which further complicates immune regulation (27). While B cell-derived antibodies against periodontal pathogens can be protective, immune complexes and autoantibodies may also contribute to chronic inflammation and tissue damage. Periodontal lesions frequently contain lymphoid follicle-like structures that sustain chronic B cell activation and perpetuate inflammation (28). Proteomic analyses have identified upregulation of Marginal Zone B and B-1 Cell-Specific Protein (MZB1), a protein involved in B cell development and antibody production, in periodontitis tissues, suggesting dysregulated B cell responses (16). Furthermore, microRNAs modulating T and B cell functions have been implicated in periodontitis pathogenesis, offering potential diagnostic and therapeutic targets (28). Overall, the dynamic interplay between effector and regulatory adaptive immune cells critically determines the balance between periodontal tissue destruction and repair, underscoring the importance of targeted immunomodulation in disease management. As **Figure 4** shown, we could easily understand how the adaptive immune cells activated and regulated.

**Figure 4:**
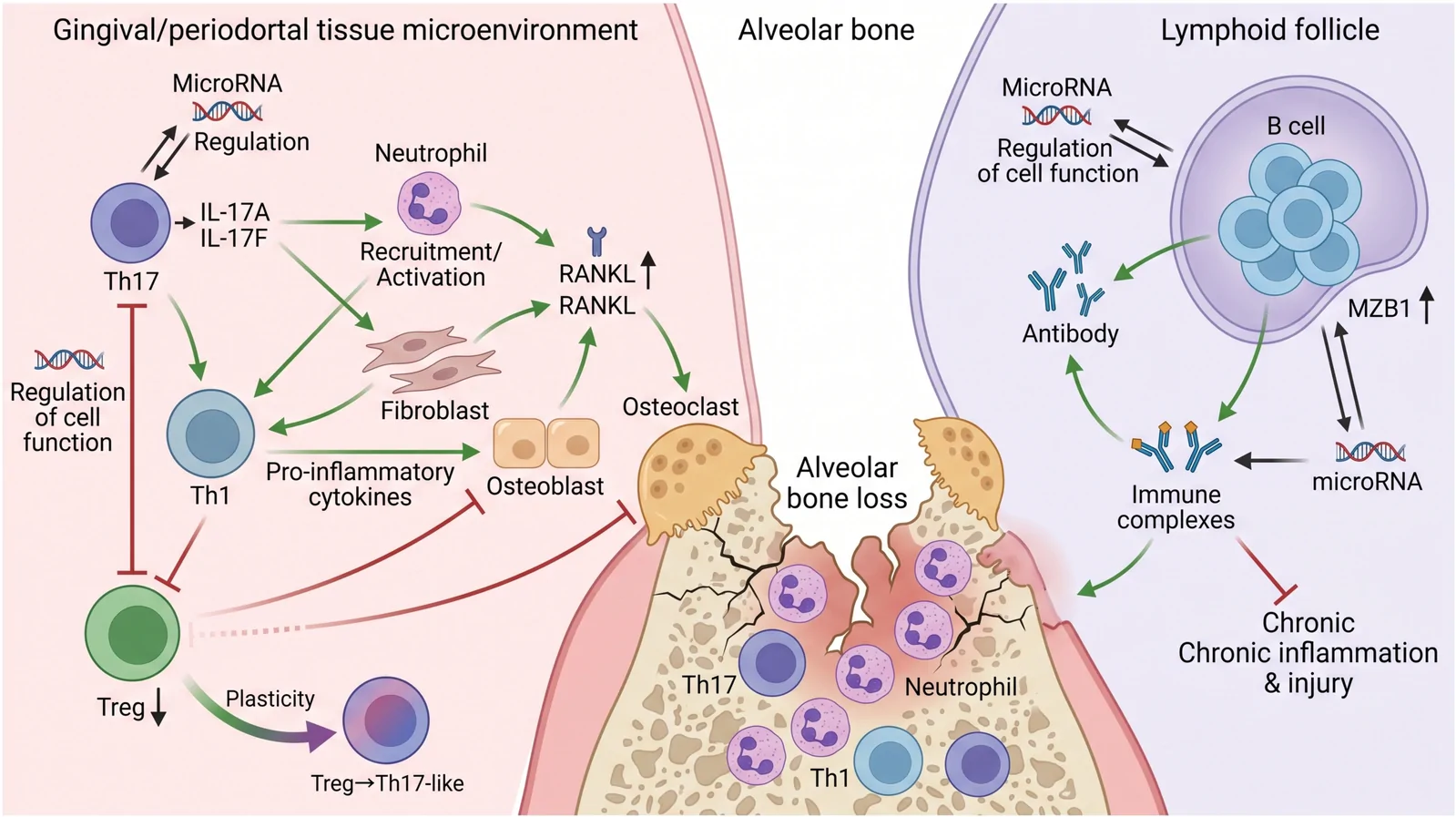
Activation and regulation of adaptive immune cells.

## 3 Molecular and cellular mechanisms by which activated inflammatory cells drive bone destruction

### 3.1 Core regulation of the RANKL/RANK/OPG axis

The RANKL/RANK/OPG axis serves as a pivotal regulatory pathway in periodontal bone destruction, orchestrating osteoclast differentiation and activation. In periodontitis, activated immune cells including T cells, B cells, and periodontal ligament fibroblasts emerge as principal sources of RANKL expression. Pro-inflammatory cytokines secreted by these inflammatory cells—such as TNF-α, IL-1β, IL-6, and IL-17—these cytokines primarily act on osteoblasts, potently upregulate RANKL expression while concurrently downregulating osteoprotegerin (OPG), a decoy receptor that inhibits RANKL-RANK interaction. OPG is a soluble receptor produced by osteoblasts and other stromal cells; it binds to RANKL with high affinity and prevents RANK activation, thereby suppressing osteoclastogenesis. In periodontitis, the downregulation of OPG by pro-inflammatory cytokines further elevates the RANKL/OPG ratio, exacerbating bone resorption (29, 31). This cytokine-driven modulation results in a markedly elevated RANKL/OPG ratio, tipping the balance toward osteoclastogenesis and bone resorption. Mechanistically, RANKL binds directly to its receptor RANK on osteoclast precursors, triggering downstream signaling cascades including NF-κB, MAPK, and Nuclear Factor of Activated T-Cells, Cytoplasmic 1(NFATc1) pathways, which are essential for osteoclast differentiation and activation. These pathways are also central to inflammatory cell activation in other diseases, where their inhibition reduces cytokine production and tissue damage (29–31), These pathways are also central to inflammatory cell activation in other diseases, where their inhibition reduces cytokine production and tissue damage. Membrane-bound RANKL (mRANKL) is a type II transmembrane protein primarily expressed on the surface of various cells through direct cell-cell contact, such as activated T cells, B cells, and osteoblasts. soluble RANKL (sRANKL) can be generated by proteolytic cleavage of mRANKL; for example, tumor necrosis factor-α converting enzyme can cleave mRANKL on the surface of activated lymphocytes to form sRANKL. Additionally, certain cells (such as myeloma cells and cervical cancer cells) can directly secrete sRANKL, while osteoblasts express both forms under inflammatory stimulation. The cleavage of mRANKL to sRANKL by TNF-a Converting Enzyme (TACE) is enhanced in inflammatory microenvironments, where activated inflammatory cells release proteases and cytokines (29, 32). sRANKL diffuses through the extracellular matrix and activates RANK on distant osteoclast precursors, thereby amplifying bone resorption beyond the immediate site of inflammation and contributing to more widespread alveolar bone loss. In contrast, mRANKL-mediated signaling requires direct cell-cell contact and is spatially restricted, which may allow for more localized osteoclast activation. This distinction has therapeutic implications: selectively blocking sRANKL could attenuate systemic bone loss while preserving the local immune functions mediated by mRANKL (32) The differential roles of mRANKL and sRANKL in alveolar bone resorption have been extrapolated from general RANKL biology, as direct evidence specific to periodontitis is limited, and selectively blocking sRANKL to attenuate systemic bone loss while preserving local immune functions mediated by mRANKL. Recent studies have further elucidated the complexity of RANKL signaling in periodontitis by distinguishing between “cis” and “trans” modes of RANKL signal transduction, suggesting that soluble RANKL may engage in distinct signaling dynamics compared to membrane-bound forms. This insight has spurred the development of targeted therapeutic strategies aiming to selectively block soluble RANKL, thereby attenuating osteoclast activation without broadly suppressing immune function (33).Experimental interventions such as Tenacissoside H and protocatechualdehyde have demonstrated efficacy in inhibiting RANKL-induced osteoclastogenesis by suppressing NF-κB and MAPK signaling pathways, reducing inflammatory cytokine production, and mitigating oxidative stress, which collectively contribute to alveolar bone preservation in inflammatory osteolysis models (34, 35). The involvement of oxidative stress and inflammatory cell activation in bone destruction is further supported by studies showing that modulation of these pathways through agents such as V-set and immunoglobulin domain-containing protein 4(VSIG4) or High Mobility Group Protein B1(HMGB1) inhibitors can attenuate tissue injury in other inflammatory conditions (36, 37).Moreover, osteocytes, traditionally considered passive bone cells, have been identified as significant contributors to RANKL production under inflammatory conditions via MYD88-dependent pathways, further amplifying osteoclastogenesis in periodontitis (3). The interplay of these cellular sources and molecular signals underscores the centrality of the RANKL/RANK/OPG axis in periodontal bone destruction and highlights the therapeutic potential of modulating this axis to restore bone homeostasis in periodontitis. And the proposed mechanism is depicted in **Figure 5**.

**Figure 5:**
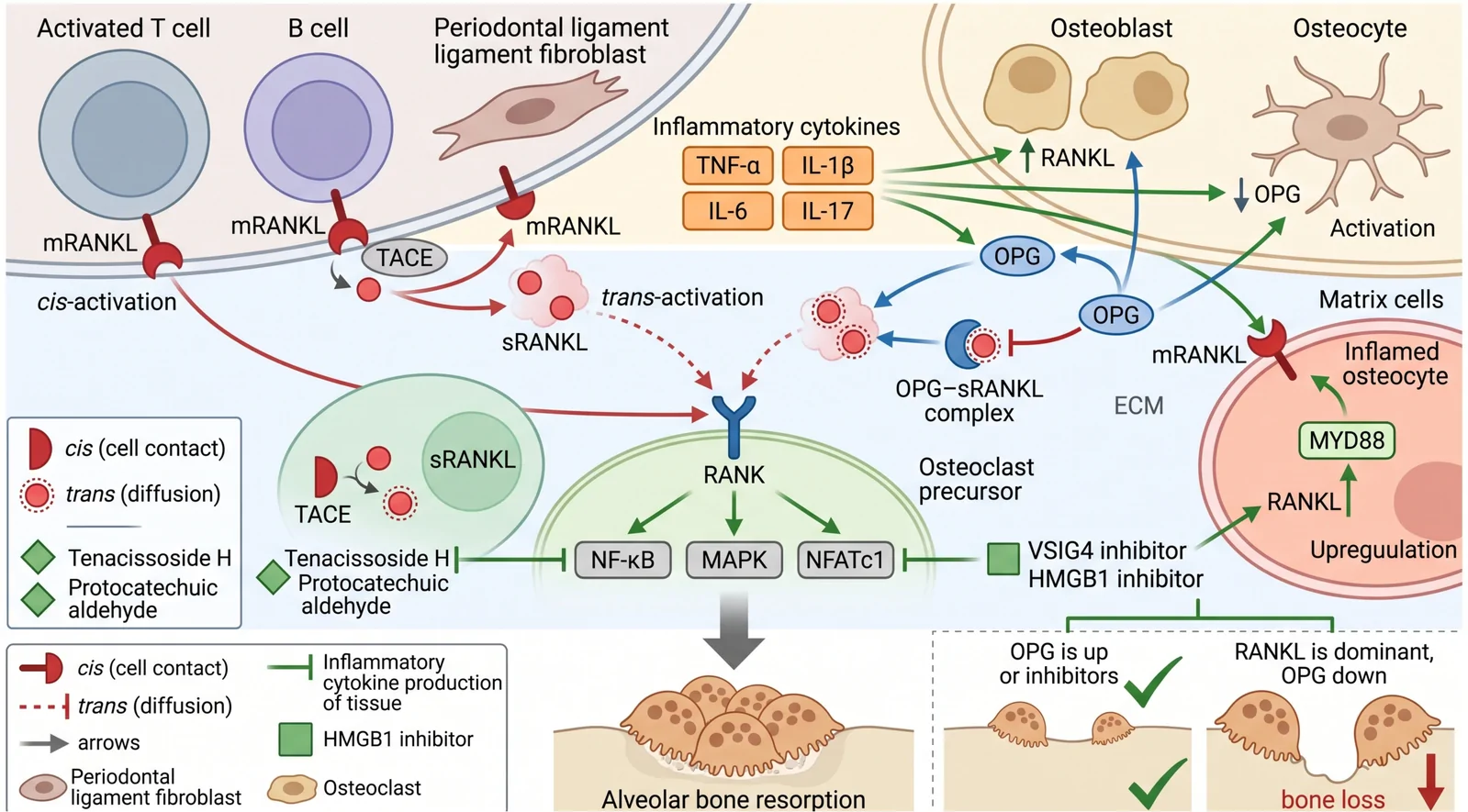
Core regulation of the RANKL/RANK/OPG axis.

### 3.2 Inflammatory cytokine-mediated osteoclastogenesis and activation

Inflammatory cytokines critically mediate osteoclastogenesis and activation in periodontal bone destruction, acting both synergistically with and independently of RANKL. TNF-α exemplifies this dual role; it not only synergizes with RANKL to robustly promote osteoclast differentiation but also, at low RANKL concentrations, independently induces osteoclastogenesis via activation of Tumor Necrosis Factor Receptor-Associated Factor 2/5(TRAF2/5)-mediated NF-κB and JNK/AP-1 signaling pathways. This capacity of TNF-α to bypass the canonical RANKL requirement underscores its potent osteoclastogenic influence in inflammatory milieus. Similarly, IL-1β amplifies RANKL-induced osteoclastogenesis by engaging its receptor to activate the MyD88/IRAK/TRAF6 signaling axis, which enhances NFATc1 expression—a master transcription factor for osteoclast differentiation—and augments the bone-resorptive activity and survival of mature osteoclasts. IL-17, predominantly produced by Th17 cells, exerts its osteoclastogenic effects indirectly by stimulating stromal cells such as osteoblasts and fibroblasts to secrete colony-stimulating factors including Granulocyte-Macrophage Colony-Stimulating Factor (GM-CSF) and Granulocyte Colony-Stimulating Factor (G-CSF). These factors expand the pool of osteoclast precursors and synergize with other cytokines to establish a pro-osteoclastogenic microenvironment. Experimental blockade of IL-17 signaling via sustained local delivery of neutralizing antibodies has demonstrated significant attenuation of alveolar bone loss and osteoclastic activity in murine periodontitis models, highlighting IL-17’s critical role in periodontal bone resorption (1). In a study of gingival tissue from patients with chronic periodontitis, the infiltration of Th17 cells was significantly associated with an elevated RANKL/OPG ratio (25). Furthermore, inflammatory cytokines contribute to oxidative stress and mitochondrial dysfunction in osteoclast precursors, as evidenced by studies on PINK1 deficiency, which exacerbates osteoclastogenesis through impaired mitophagy and increased ROS production, thereby enhancing NFATc1 signaling and bone resorption (4). Collectively, these inflammatory mediators orchestrate a complex network that promotes osteoclast differentiation, activation, and survival, driving the pathological bone loss characteristic of periodontitis. As schematically illustrated in **Figure 6**, how inflammatory cytokine-mediated osteoclastogenesis and activation worked.

**Figure 6:**
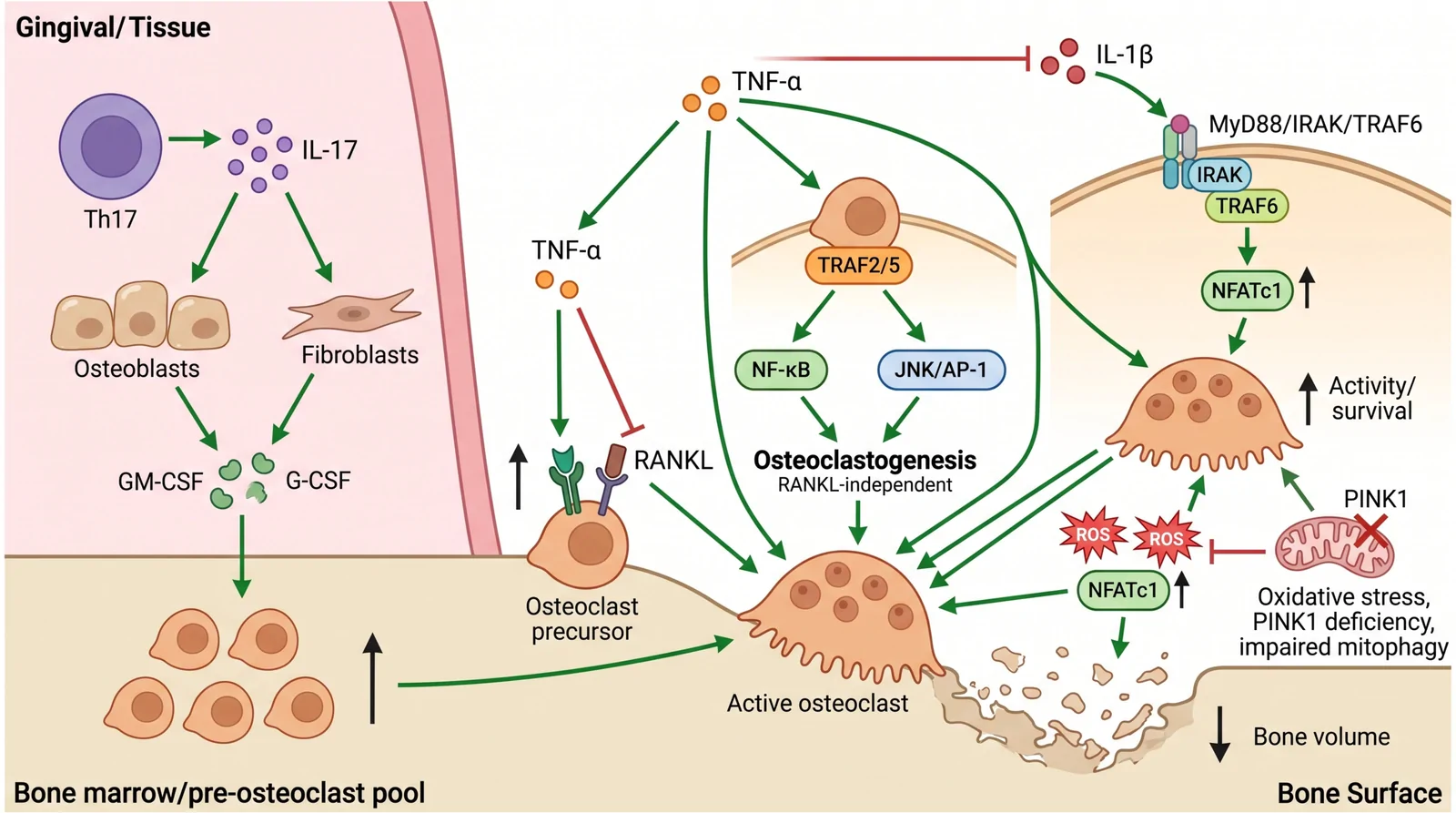
Inflammatory cytokine-mediated osteoclastogenesis and activation.

### 3.3 Interactions between inflammatory cells and bone cells: osteocytes and osteoblasts

The crosstalk between inflammatory cells and bone-resident cells—osteocytes and osteoblasts—plays a decisive role in the dysregulation of bone remodeling during periodontitis. Osteocytes, embedded within the mineralized bone matrix, function as mechanosensors and endocrine regulators of bone homeostasis. Under inflammatory stimuli, osteocytes undergo apoptosis and upregulate expression of RANKL and sclerostin (SOST), thereby initiating and amplifying osteoclastic bone resorption. The MYD88-dependent signaling pathway in osteocytes has been identified as a critical mediator of this process, with bacterial pathogen-associated molecular patterns inducing RANKL expression and consequent osteolysis (3). Concurrently, activated macrophages and T cells secrete pro-inflammatory cytokines such as the same pro-inflammatory cytokines discussed above, which inhibit osteoblast differentiation and mineralization while promoting osteoblast apoptosis. This suppression of osteoblastic bone formation disrupts the coupling between bone resorption and formation, favoring net bone loss. Emerging evidence highlights extracellular vesicles, particularly exosomes derived from M1-polarized macrophages, as novel mediators of intercellular communication in this context (38). These vesicles carry microRNAs such as miR-155 that suppress osteogenic gene expression in osteoblasts, further impairing bone formation. Therapeutic strategies targeting these vesicle-mediated pathways or restoring osteoblast function have shown promise in experimental models. Additionally, inflammatory conditions induce premature senescence of alveolar osteocytes, characterized by increased expression of senescence markers and a senescence-associated secretory phenotype (SASP) that exacerbates local inflammation and bone resorption (37). Thus, the dynamic interactions between inflammatory cells and bone cells create a vicious cycle of enhanced osteoclastogenesis and impaired osteogenesis, culminating in the alveolar bone destruction observed in periodontitis. Understanding these cellular dialogues provides critical insights for developing targeted interventions to restore bone remodeling balance and prevent periodontal bone loss. **Figure 7** shows the interactions between inflammatory cells and bone cells.

**Figure 7:**
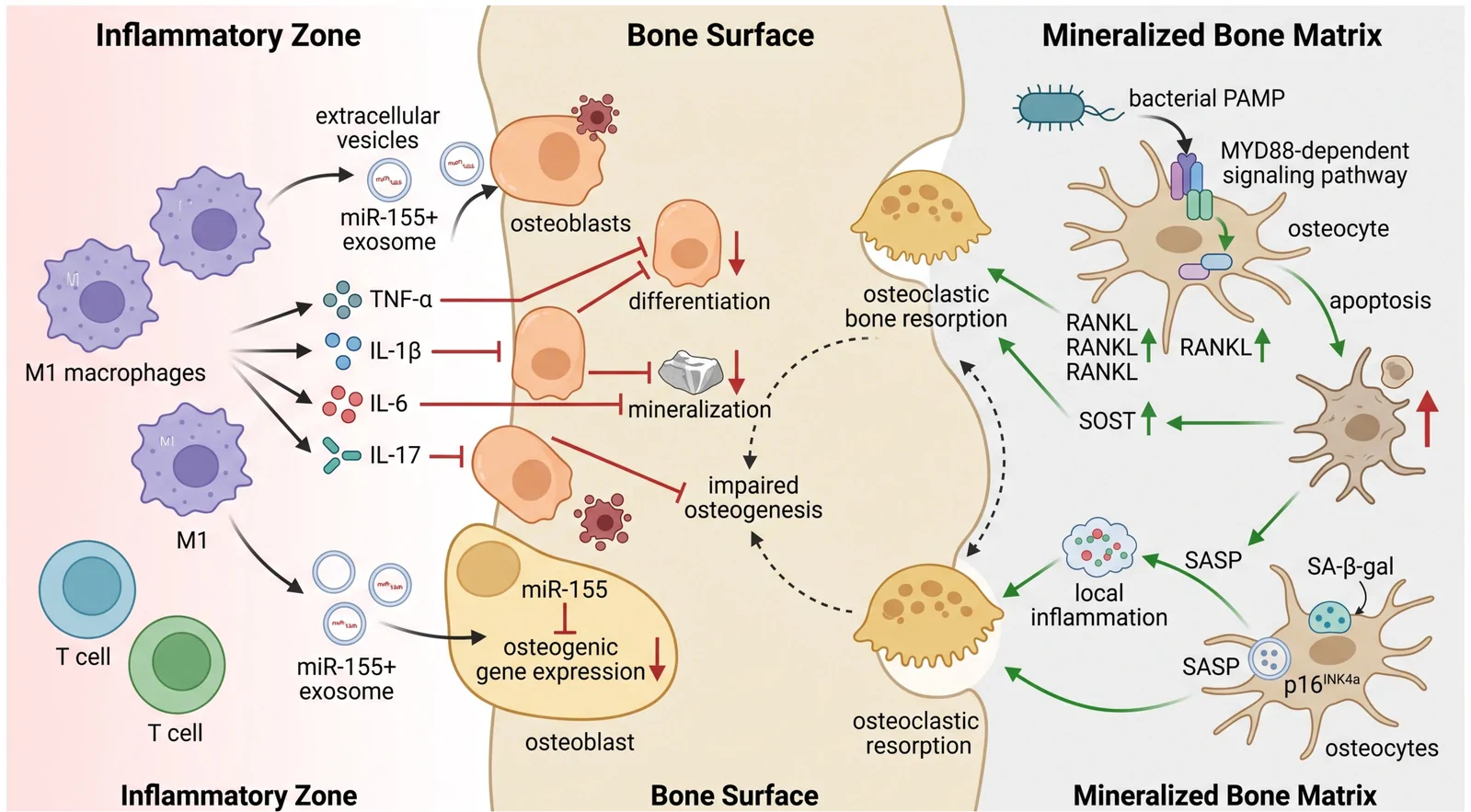
Interactions between inflammatory cells and bone cells: osteocytes and osteoblasts.

## 4 Intervention strategies and research progress targeting inflammatory cell activation

### 4.1 Small molecule inhibitors and signal pathway blockers

Small molecule inhibitors targeting key inflammatory signaling pathways have emerged as promising therapeutic agents to mitigate periodontal inflammation and alveolar bone destruction. Among these, inhibitors of the Inhibitor of Kappa B Kinase (IKK)/NF-κB pathway, anus kinase and signal transducer and activator of transcription (JAK/STAT) pathway, and p38 MAPK pathway have demonstrated efficacy in animal models of periodontitis. The NF-κB pathway is a central regulator of inflammatory gene expression, and its inhibition reduces pro-inflammatory cytokines and osteoclastogenic factors, thereby attenuating bone resorption. For instance, natural compounds such as avenanthramide-C have been shown to suppress NF-κB activation and downstream catabolic factors like MMP1 and IL-6 in gingival fibroblasts, leading to reduced alveolar bone erosion in ligature-induced periodontitis models (39). Similarly, p38 MAPK inhibitors decrease inflammatory mediator production and osteoclast differentiation, further protecting alveolar bone. JAK/STAT inhibitors, by modulating cytokine signaling, also reduce periodontal inflammation and bone loss, although specific studies in periodontitis models remain limited.

Targeting the RANKL pathway, which is pivotal for osteoclast differentiation and activation, has been a cornerstone in managing bone resorption. Denosumab, a monoclonal antibody against RANKL, is clinically approved for osteoporosis and has been investigated for aggressive periodontitis. Denosumab is clinically approved for osteoporosis, but its use in periodontitis remains experimental, with limited human data and concerns about osteonecrosis of the jaw with long-term systemic use. However, translational studies reveal that RANKL inhibition alone may not fully prevent bacterial osteolysis, as bone destruction in infection involves multifactorial mechanisms including proteolysis and neutrophil activity (40). Nonetheless, local administration of osteoclast inhibitors such as reveromycin A ointment has shown promising results in experimental periodontitis by reducing osteoclast numbers and alveolar bone loss (41). These findings suggest that small molecule inhibitors targeting osteoclastogenesis can be effective adjuncts in periodontal therapy.

Another critical target is the NLRP3 inflammasome, which mediates maturation of IL-1β and IL-18, key cytokines in periodontal inflammation. Inhibitors like MCC950 suppress inflammasome activation in macrophages and neutrophils, reducing inflammatory cytokine release and alveolar bone destruction in experimental periodontitis models (2). Given the inflammasome’s role in amplifying inflammation and osteoclast activity, its inhibition represents a novel therapeutic avenue. Moreover, modulation of leukotriene signaling with antagonists such as montelukast has demonstrated decreased alveolar bone loss and inflammation in rat periodontitis models, highlighting the potential of targeting lipid mediator pathways in inflammation and alveolar bone loss (42).

Collectively, these small molecule inhibitors and signaling pathway blockers offer multifaceted approaches to dampen periodontal inflammation and osteoclast-mediated bone resorption. Their efficacy in preclinical models underscores the importance of targeting both inflammatory cascades and osteoclastogenesis. However, clinical translation requires careful consideration of systemic effects, dosing, and delivery methods to maximize local efficacy while minimizing adverse effects. Future research integrating these inhibitors with conventional periodontal therapies may enhance treatment outcomes and preserve alveolar bone integrity.

Despite their promise, the clinical translation of small-molecule inhibitors is hindered by several limitations. Systemic exposure to agents targeting NF-κB, p38 MAPK, or JAK/STAT may cause off-target immunosuppression, hepatotoxicity, or gastrointestinal adverse effects, and achieving therapeutically relevant concentrations within periodontal pockets while avoiding systemic toxicity remains a major delivery challenge. This has prompted the development of locally or inhaled-administered inhibitors—for example, inhaled Phosphodiesterase 4(PDE4) inhibitors for Chronic Obstructive Pulmonary Disease (COPD)—to reduce systemic exposure and adverse reactions (30), which directly informs the design of locally administered small-molecule inhibitors for periodontitis, such as gingival crevicular fluid-targeted formulations. Furthermore, pathway redundancy and context-dependent functions can dampen efficacy; thrombospondin-1, for instance, exhibits both pro- and anti-inflammatory roles depending on the microenvironment, illustrating that single-target blockade may yield unpredictable outcomes (43). Currently, most of these agents are at the preclinical proof-of-concept or early clinical stage, and only a few have advanced to phase II/III trials. Notably, the phase II/III trials reported for these agents are for indications other than periodontitis (e.g., rheumatoid arthritis, cancer), while their application in periodontitis remains at preclinical or early clinical stages. Thus, careful optimization of target specificity, dosing, and local delivery is essential before these inhibitors can be translated to periodontitis therapy. As schematically illustrated in **Figure 8**, we could see small molecule Inhibitors and signal pathway blockers work at different inflammatory cells.

**Figure 8:**
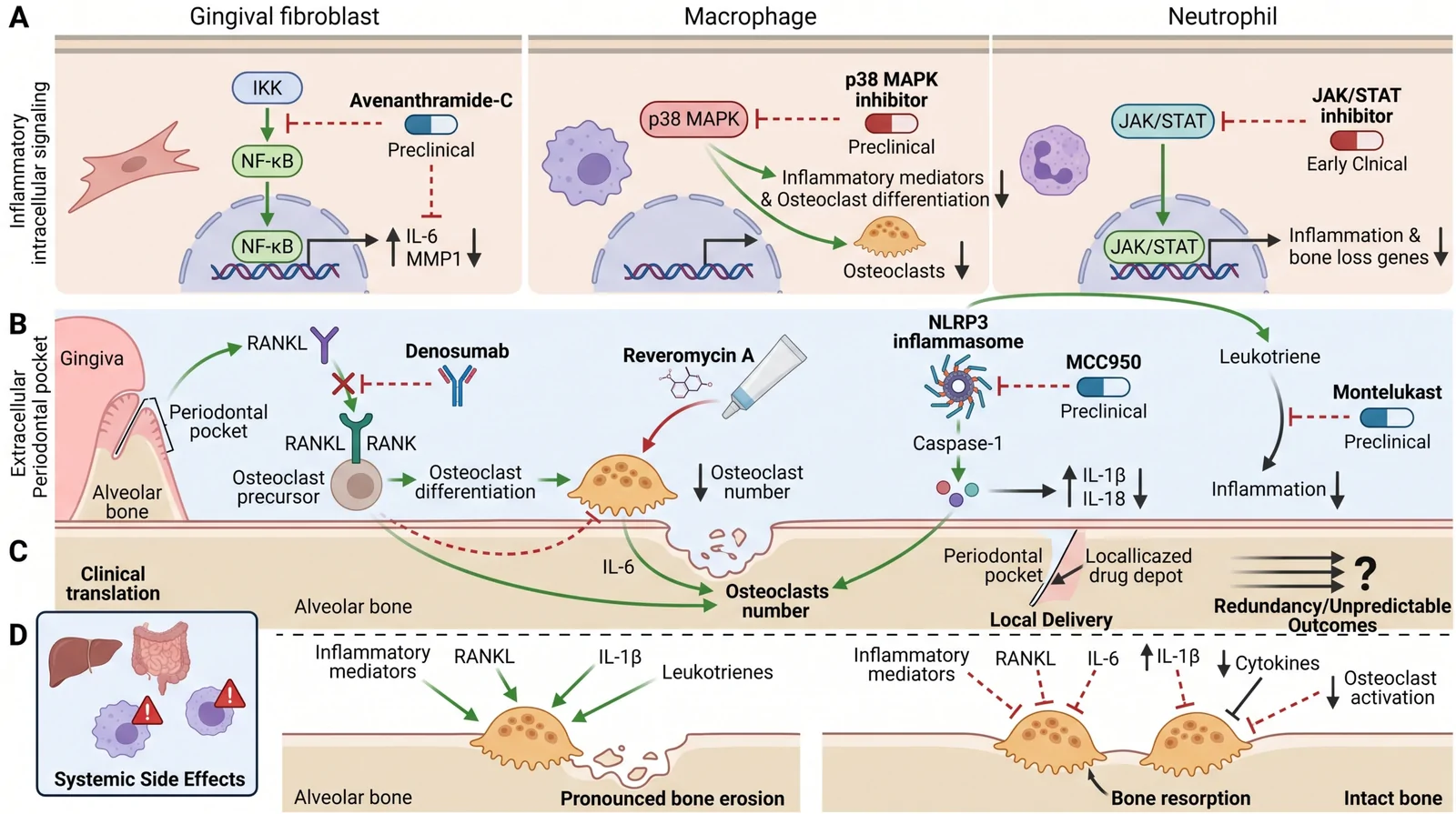
Potential therapeutic effects of small molecule inhibitors and signaling pathway blockers on periodontal inflammation and alveolar bone destruction. **(A)** Intracellular signal transduction of inflammatory mediators. **(B)** Small molecule inhibitors and signaling pathway blockers have demonstrated efficacy in reducing periodontal inflammation and alveolar bone resorption in preclinical models. **(C)** Unpredictability faced by small molecule inhibitors and signaling pathway blockers in clinical translation. **(D)** Possible systemic side effects of small molecule inhibitors and signaling pathway blockers.

### 4.2 Biologics and antibody therapies

Biologic agents and antibody therapies targeting pro-inflammatory cytokines have revolutionized treatment paradigms in systemic inflammatory diseases and are increasingly explored for refractory periodontitis, especially in patients with comorbidities. Tumor necrosis factor-alpha (TNF-α) inhibitors such as adalimumab, interleukin-1β (IL-1β) antagonists like canakinumab, and interleukin-6 receptor (IL-6R) blockers such as tocilizumab have established efficacy in rheumatoid arthritis and other autoimmune conditions. One study suggests that even for severe intraosseous defects with limited regenerative potential, successful periodontal tissue regeneration can be achieved through the combined use of materials such as biologic agents, thereby indirectly reducing inflammation and promoting alveolar bone repair (44). Their immunomodulatory effects can attenuate the exaggerated host response that drives periodontal tissue destruction.

Targeting the IL-17A/IL-17RA axis with monoclonal antibodies directly interferes with the Th17 cell-mediated inflammatory pathway, which is central to periodontal pathogenesis. Animal studies demonstrate that IL-17 blockade effectively suppresses alveolar bone resorption and inflammatory cytokine production, positioning IL-17 as a promising therapeutic target (45). Human clinical data on IL-17 inhibitors in periodontitis are currently lacking, But it given the pivotal role of Th17 cells in orchestrating osteoclastogenesis and neutrophil recruitment, IL-17 inhibitors may provide targeted control of periodontal inflammation and bone loss.

Innovative approaches involving bispecific antibodies or fusion proteins are being developed to simultaneously target multiple pathogenic mediators. For example, dual targeting of TNF-α and RANKL aims to concurrently suppress inflammation and osteoclast activation, potentially enhancing therapeutic efficacy. Additionally, antibody-drug conjugates designed to recognize specific activation markers on immune cells could deliver cytotoxic agents selectively to pathogenic inflammatory cells, minimizing systemic immunosuppression. Although these strategies are in early stages, they represent a frontier for precision immunotherapy in periodontitis.

Despite their potential, biologics pose challenges including high cost, risk of systemic immunosuppression, and the need for parenteral administration. Moreover, the heterogeneity of periodontitis and its multifactorial etiology necessitate careful patient selection and monitoring. Integration of biologics with conventional mechanical debridement and local therapies may optimize outcomes. Ongoing clinical trials and translational research are essential to establish safety, efficacy, and protocols for biologic use in periodontal disease, particularly in patients with systemic inflammatory comorbidities.

Biologics and antibody therapies face additional barriers in periodontitis. Their high molecular weight limits penetration into gingival tissue and alveolar bone, and parenteral administration is impractical for a chronic local disease. Long-term systemic immunosuppression increases the risk of infection and may interfere with protective immune responses. Moreover, the heterogeneity of inflammatory cell activation in periodontal lesions means that blocking a single cytokine or pathway may not restore homeostasis: in atherosclerosis research, oxidized low-density lipoprotein (oxLDL)-driven inflammatory cell activation is not directly targeted by any current anti-inflammatory agent, reflecting the gap between mechanistic targets and effective therapeutic translation (29),which demonstrated that blocking a single pathway may not restore homeostasis in heterogeneous inflammatory micro-environments, a challenge applicable to periodontitis where diverse immune cell subsets are activated. Similarly, persistent blood-ocular barrier dysfunction in uveitis can remain despite apparent control of inflammatory cell activity, leading to discordant clinical findings and relapse (46),which highlighted the discordance between cellular activity and clinical outcomes, which parallels the risk of incomplete resolution of periodontal tissue destruction despite anti-inflammatory therapy. In Inflammatory Bowel Disease (IBD), B-cell-targeted therapies show variable efficacy across patient subgroups, underscoring the need for biomarker-guided patient selection (47), underscored the importance of biomarker-guided patient stratification, which is demonstrated for periodontitis given its heterogeneous clinical phenotype. Preclinical studies of recombinant thrombomodulin in chronic graft-versus-host disease demonstrate reduced inflammatory cell recruitment, but clinical evidence is still limited (48), which demonstrated reduced inflammatory cell recruitment, a concept that could be adapted to periodontitis by targeting local endothelial activation to limit leukocyte infiltration. Therefore, biologic therapies for periodontitis are at various stages, spanning preclinical animal models to early clinical trials, and robust safety/efficacy data are required before clinical adoption. The proposed mechanism is depicted in **Figure 9**, that shows how the biologics and antibody therapies work.

**Figure 9:**
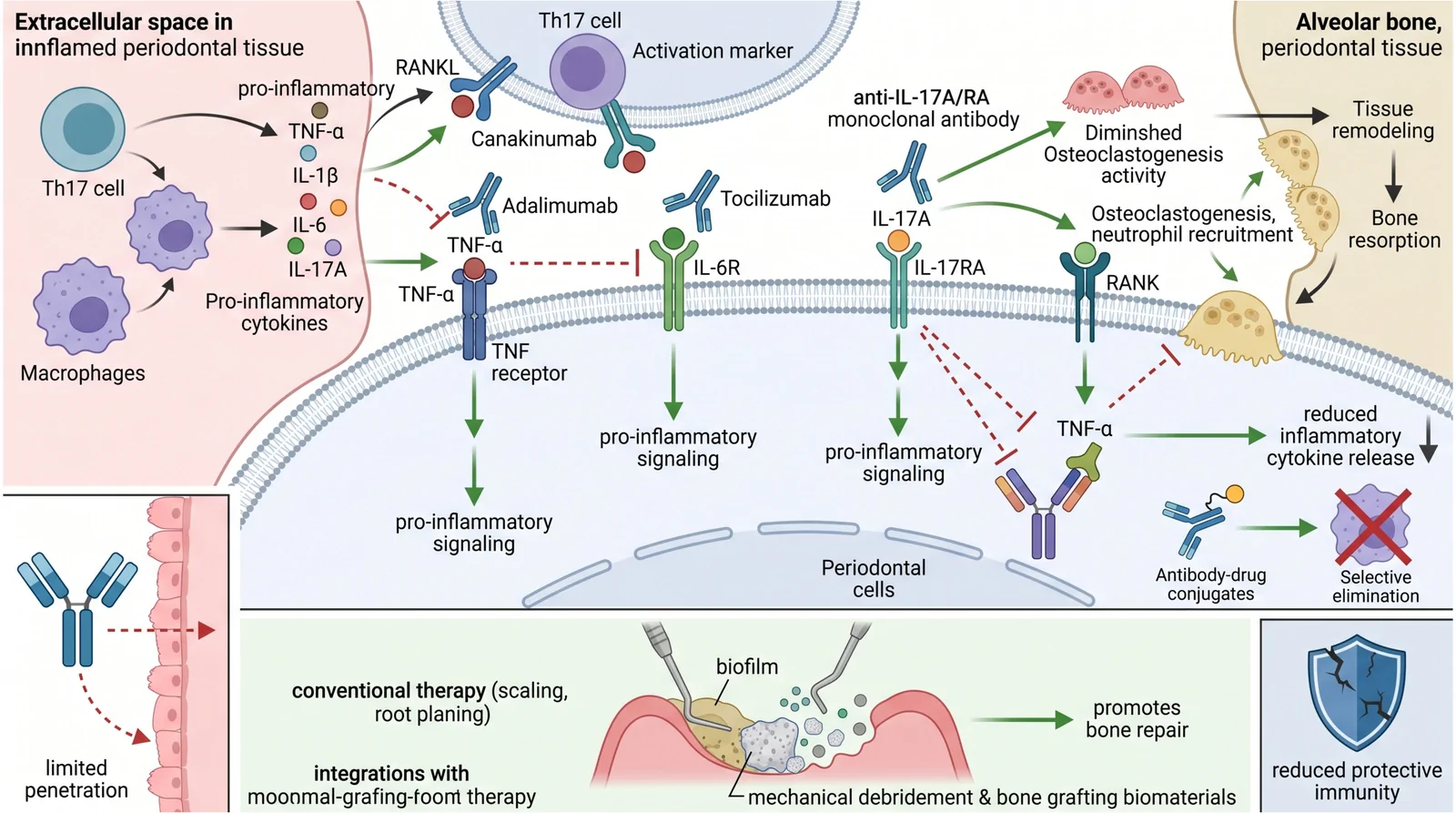
Biologics and antibody therapies.

### 4.3 Cell therapy and immunomodulatory strategies

Cell-based therapies and immunomodulation represent cutting-edge approaches to restore periodontal tissue homeostasis by harnessing the regenerative and anti-inflammatory capacities of specific cell populations. Mesenchymal stem cells (MSCs) have garnered significant attention due to their potent immunoregulatory properties and ability to differentiate into osteoblasts, facilitating alveolar bone regeneration. MSCs modulate immune responses via direct cell-cell interactions and secretion of paracrine factors such as prostaglandin E2 (PGE2), indoleamine 2,3-dioxygenase (IDO), and tumor necrosis factor-stimulated gene-6 (TSG-6). These mediators suppress T and B lymphocyte proliferation, promote macrophage polarization toward the anti-inflammatory M2 phenotype, and enhance osteogenic differentiation, collectively contributing to reduced inflammation and bone repair in periodontitis (49).

Adoptive transfer of regulatory T cells (Tregs) or pharmacological expansion of endogenous Tregs using low-dose interleukin-2 or rapamycin offers another immunomodulatory avenue. Tregs restore immune tolerance in the periodontal microenvironment, suppressing excessive inflammatory responses that drive tissue destruction. Animal models demonstrate that Treg-based therapies effectively inhibit experimental periodontitis progression by dampening pro-inflammatory cytokine production and osteoclast activation (50). These strategies aim to recalibrate the immune balance rather than broadly suppress immunity, potentially reducing side effects. To further improve safety and efficacy, advanced delivery systems are being developed to localize these immunomodulatory effects.

Advanced delivery systems employing nanocarriers or hydrogels enable localized, controlled release of anti-inflammatory cytokines such as interleukin-10 (IL-10), microRNA mimics, or inhibitors targeting pro-inflammatory microRNAs like miR-155. Such spatiotemporal precision in modulating inflammatory cell activation within periodontal pockets enhances therapeutic efficacy while minimizing systemic exposure (50). For example, injectable thermosensitive hydrogels loaded with natural compounds like bakuchiol have demonstrated antibacterial and anti-inflammatory effects, reducing alveolar bone loss in rat periodontitis models (51).

Collectively, cell therapy and immunomodulatory strategies offer promising avenues to not only halt periodontal inflammation but also promote tissue regeneration. Challenges remain in optimizing cell sources, delivery methods, and ensuring long-term safety and efficacy. Integration with existing periodontal treatments and personalized approaches based on patient immune profiles may maximize therapeutic benefits. Continued preclinical and clinical investigations are crucial to translate these innovative therapies into routine clinical practice for managing periodontitis and preserving alveolar bone health.

Cell-based and immunomodulatory strategies must overcome substantial translational hurdles. Mesenchymal Stem Cell (MSC) production requires Good Manufacturing Practice (GMP)-compliant manufacturing, rigorous quality control, and the choice between autologous and allogeneic sources; they’re *in-vivo* survival and engraftment remain difficult to predict. Advanced delivery systems, such as *in-situ* forming hydrogel coatings that inactivate inflammatory cells, have been tested in blood-contacting devices and clinical sepsis patients, illustrating the feasibility of local immunomodulation, but these systems have not yet been adapted to periodontitis (52). Preclinical studies have shown that targeting S100a9 in pulmonary arterial hypertension (53) or VSIG4 in intracranial hemorrhage (36)reduces inflammatory cell activation in rodent models; which could be translated to periodontal pocket delivery systems, however, these approaches are at the proof-of-concept stage. NAMO also demonstrated protective effects in sepsis-induced acute lung injury in mice, but clinical validation is lacking (54). A topical magnetized saline water hydrogel, which promotes healing through autophagy and modulation of inflammatory cell activation, has been evaluated in a case series of elderly patients with pressure ulcers, providing early clinical evidence for such local immunomodulatory devices (55). Nevertheless, most cell therapies and immunomodulatory strategies remain at preclinical or early clinical stages, and production scalability, delivery efficiency, and long-term safety are critical issues to address for eventual application in periodontitis. Here, we propose a working model (**Figure 10**) in which cell therapy and immunomodulatory strategies might be.

**Figure 10:**
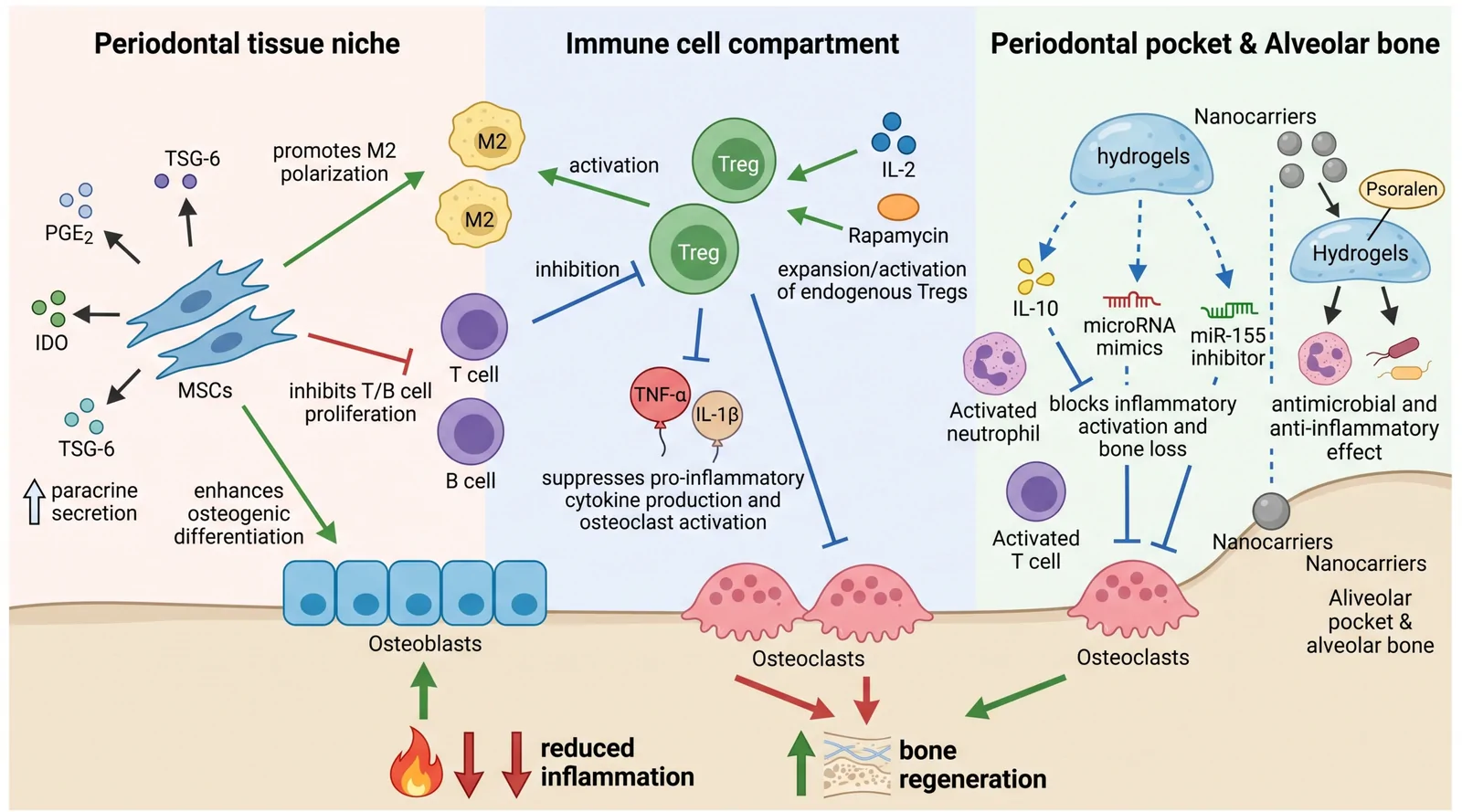
Cell therapy and immunomodulatory strategies.

## 5 Conclusion

Periodontal disease (PD) is a chronic inflammatory condition driven by oral microbial dysbiosis, where a polymicrobial community shifts toward pathogenic bacteria, particularly the “red complex” triad (Porphyromonas gingivalis, Tannerella forsythia, and Treponema denticola), which synergistically promote disease progression through virulence factors and biofilm formation (56–58).These dysbiotic pathogens initiate host immune activation primarily through pattern recognition receptors (PRRs), such as Toll-like receptors (TLRs), notably TLR4, which recognize pathogen-associated molecular patterns (PAMPs, 57, 59). This PAMP-PRR signaling triggers downstream pro-inflammatory cascades, including NF-κB activation, leading to the recruitment and activation of immune cells (e.g., macrophages and neutrophils) and the subsequent release of pro-inflammatory cytokines (IL-1, IL-6, TNF-α) and prostaglandins (PGE2, 57). These inflammatory mediators are critical drivers of immune cell-mediated alveolar bone destruction, a hallmark of periodontitis severity, as evidenced by the strong correlation between elevated bacterial loads (especially red complex species) and advancing disease stages or clinical attachment loss (60, 61).

The intricate interplay of inflammatory cells stands at the forefront of periodontal bone destruction, underscoring the complexity of the disease’s pathogenesis. This review highlights how macrophages, neutrophils, T cells, and B cells collectively orchestrate a pro-osteoclastic microenvironment through a sophisticated network of cellular interactions. Central to this process is the regulation of the RANKL/RANK/OPG axis, which serves as a pivotal molecular pathway modulating osteoclastogenesis and bone resorption. The influence of various inflammatory mediators on osteoclast differentiation, activation, and osteoblastic function further complicates this landscape, revealing a delicate balance between bone destruction and formation.

Recent advances have expanded our understanding beyond traditional paradigms by uncovering the heterogeneity within inflammatory cell subsets and the emerging role of extracellular vesicles as critical modulators of intercellular communication. These findings emphasize the necessity of moving past a one-dimensional view of inflammatory cells toward appreciating their dynamic and context-dependent functions in periodontal disease. Balancing these diverse research perspectives—ranging from molecular signaling pathways to cellular heterogeneity—provides a more comprehensive framework for understanding periodontal bone loss and its progression.

Therapeutically, the field is witnessing a paradigm shift from broad-spectrum anti-inflammatory approaches to precision immunomodulation. Targeted interventions, including small molecule inhibitors, biologics, and cell-based therapies, demonstrate significant promise in modulating inflammatory cell activity with greater specificity. However, translating these innovations into routine clinical practice remains challenging. Critical issues such as target specificity, efficient local delivery, and long-term safety profiles must be rigorously addressed to ensure both efficacy and patient safety. This necessitates a multidisciplinary approach integrating immunology, pharmacology, and bioengineering to refine these therapeutic modalities.

Looking forward, the integration of multi-omics technologies offers a powerful avenue to dissect the dynamic inflammatory cell landscape across different stages of periodontitis. Such comprehensive profiling will enable the identification of distinct immune phenotypes, facilitating the development of personalized treatment strategies tailored to individual patient profiles. This precision medicine approach holds the potential not only to arrest periodontal bone destruction effectively but also to promote tissue regeneration, thereby restoring periodontal health.

In conclusion, the evolving understanding of inflammatory cell roles in periodontal bone destruction underscores the complexity and heterogeneity inherent in the disease process. By balancing mechanistic insights with translational research, the field is poised to develop more targeted and effective therapies. Continued efforts to elucidate the dynamic immune microenvironment and to harness precision immunomodulatory strategies will be essential in overcoming current therapeutic limitations. Ultimately, these advances promise to transform the management of periodontitis, shifting from symptomatic control to disease modification and regeneration, thereby relieving pain, preventing tooth loss, improving patient outcomes and quality of life.
